# Describing the leadership capabilities of advanced practice nurses using a qualitative descriptive study

**DOI:** 10.1002/nop2.150

**Published:** 2018-04-25

**Authors:** Alyson Lamb, Ruth Martin‐Misener, Denise Bryant‐Lukosius, Margot Latimer

**Affiliations:** ^1^ IWK Health Centre Halifax NS Canada; ^2^ School of Nursing Dalhousie University Halifax NS Canada; ^3^ School of Nursing & Department of Oncology McMaster University Halifax ON Canada

**Keywords:** advanced practice, ANP, leadership, nurses, nursing

## Abstract

**Aim:**

The aim of this study is to explore advanced practice nurses’ perceptions of their leadership capabilities.

**Design:**

A qualitative descriptive methodology informed by a well‐established leadership framework was used to explore advanced practice nurses’ perceptions of their leadership.

**Methods:**

Purposive sampling of advanced practice nurses working in tertiary acute care facilities in Eastern Canada was employed. Data sources included face‐to‐face interviews and document analysis. Fourteen advanced practice nurses participated in two audio‐taped semi‐structured interviews from March 2013–January 2014. Data were transcribed and analysed using NVIVO 10 software and content analysis.

**Results:**

Two main themes were identified: “Patient‐focused leadership” and “organization and system‐focused leadership”. These two themes are further described through leadership domains and capabilities that clearly articulate advanced practice nursing leadership and its contribution to improving the care environment for patients and families, nurses and other healthcare providers, organizations and the healthcare system.

## 1 INTRODUCTION

Health care is in the midst of major change, stimulated by the current economic challenges facing the world, the shifting demographics of most developed countries’ populations and the growing number of people living with long‐term illness. This unpredictable and dynamic environment requires healthcare professionals to demonstrate effective leadership.

Registered nurses working in advanced practice nursing (APN) roles have completed graduate education, have an expert level of knowledge and complex decision‐making skills and clinical competencies for expanded practice specific to the context in which they are credentialed to work (International Council of Nurses, 2009, p.1; National Council of State Boards of Nursing, 2008). Advanced practice nursing roles vary by title and description between countries. The two types of APN roles formally recognized in Canada are the clinical nurse specialist and nurse practitioner. Both are clinical roles that require master's level education and expected to embody the following five competencies; clinical care, leadership, research, consultation and collaboration (Canadian Nurses Association, 2008, 2010, 2014). In Canada, clinical nurse specialists provide expert specialized clinical guidance and leadership to assist nursing staff in managing complex patients and function within the same legislative and regulatory framework as registered nurses (CNA, 2008; Donald et al., 2010). Nurse practitioners have an autonomous expanded clinical practice that includes making a medical diagnosis, ordering diagnostic tests and prescribing medications and other treatments and function within a separate nurse practitioner‐specific legislative and regulatory framework (CNA, 2008; Donald et al., 2010). Leadership is an internationally accepted generic feature of APN (Mantzoukas & Watkinson, 2007). Advanced practice nurses are expected to demonstrate leadership competencies at an advanced level in their roles (Elliot et al., 2012; Nieminen, Mannevaara, & Fagerstrom, 2011; Schober & Affara, 2006), yet there are few comprehensive explanations about what an advanced level of leadership means or how to operationalize it in healthcare environments.

Although APN roles have been in place for several decades, healthcare organization decision‐makers (managers, senior administrators and physicians) identify they have limited knowledge about the value‐added elements of these roles (Carter et al., 2013). Leadership, for example, is a capability that is often embedded in a series of complex actions in care delivery and therefore may not be apparent to decision‐makers (Calkin, 1984; Whitehead, Welch Dittman, & McNulty, 2017). This is problematic because to fully optimize the use of APN roles, clear expectations for intended outcomes must be established (Bryant‐Lukosius et al., 2007; Carter et al., 2010; Kilpatrick et al.,^2014^). The lack of knowledge about APN leadership constrains use of the full scope of these practice roles, thus limiting their potential impact on patient care and health system innovations. This article begins to address this knowledge gap by providing a clear description of APN leadership from the perspective of advanced practice nurses working in acute care settings.

### 1.1 Background

Leadership is a set of skills and abilities that a person embodies (Kouzes & Posner, 2007). A leader is the person who embodies and uses these abilities and skills to create a vision and engage others to share that vision. Although leadership is an expected attribute of all registered nurses, leadership in the profession is often considered to be role‐dependent (Scott & Miles, 2013). Those in administrative and management roles are considered formal leaders, while nurses in clinical roles, such as advanced practice nurses, are defined as informal or clinical leaders. The evidence supporting the relationship between formal nursing leadership and positive outcomes for patients and nursing staff is growing (Wong & Cummings, 2007). However, research examining the impacts of leadership demonstrated by informal nurse leaders on patients, fellow nurses and the broader system is scarce (Cummings, 2011). Advanced practice nurses are frontline healthcare providers and as such are often not recognized as formal leaders because the majority of their time is spent providing direct patient care. Although the demands of clinical practice often limit the time advanced practice nurses have for other advanced nursing practice activities, it creates opportunities to provide leadership at the frontline (Kilpatrick, DiCenso, et al., 2014; Mayo et al., 2010).

There is a substantive and growing body of synthesized research evidence demonstrating that advanced practice nurses provide safe and effective clinical care to patients (Kilpatrick et al., 2015; Martin‐Misener et al., 2015; Newhouse et al., 2011). Clinical care is the central core of APN roles, but it is only one of several dimensions that make these roles advanced. Research describing dimensions other than clinical practice such as leadership is needed if those aspects of APN roles are to be integrated and supported in practice (Bryant‐Lukosius, DiCenso, Browne, & Pinelli, 2004; Pauly et al., 2004). When advanced practice nurses are unable to fulfil all dimensions of their role, they are less satisfied and more likely to leave the role (Bryant‐Lukosius et al., 2007; Kilpatrick, DiCenso, et al., 2014).

Research about APN leadership is limited and mostly focused on elements related to team effectiveness and evidence‐based practice (Campbell & Profetto‐McGrath, 2013; Kilpatrick et al., 2013; van Soeren, Hurlock‐Chorrstecki, & Reevesm, 2011). An exception is a large case study, conducted in 13 sites across Ireland and involving 23 Clinical Nurse Specialists and Advanced Practitioners (Clinical Midwife Specialists and Advanced Nurse Practitioners). An important finding of this study was the identification of multiple activities that focused on two areas of leadership; clinical and professional (Begley et al., 2013; Elliot, Begley, Kleinpell, & Higgins, 2013; Elliot et al., 2012). Clinical leadership activities supported the development of nursing practice in clinical settings or services. Professional leadership activities supported the development of nursing practice outside the service at the national or international level (Elliot et al., 2013, p. 1039).

There are not any similar comprehensive studies of APN leadership in Canada. Differences in APN leadership may occur across countries due to the variation in role development, education, health policies and deployment. The ability to clearly identify and articulate APN leadership capabilities and their impact is needed, so that advanced practice nurses can be optimally educated and deployed to close the current leadership gap in healthcare organizations (Cummings & Estabrooks, 2003; Dickson, Briscoe, Fenwick, MacLeod, & Romilly, 2007; McCutcheon, Doran, Evans, McGillis Hall, & Pringle, 2009). The primary research question of this study was: How do advanced practice nurses working in an acute care setting describe their leadership and its impact on patient, healthcare providers and healthcare system outcomes?

## 2 THE STUDY

### 2.1 Design

The study was conducted using qualitative description (Sandelowski, 2000, 2010) informed by the LEADS in a Caring Environment Leadership Capabilities Framework, (LEADS, http://www.leadersforlife.ca/). LEADS is particularly relevant to this study because it was developed and is used by Canadian healthcare organizations, providing a common and coordinated approach to developing high quality healthcare leaders (Dickson et al., 2007).

### 2.2 Methods

The study was conducted in two tertiary acute care facilities in Eastern Canada. Advanced practice nurses received an email invitation from a senior administrator in their facility. The invitation described the study and who to contact if they were interested in participating. Recruitment posters were also placed in the two facilities.

Purposeful sampling was used to select advanced practice nurses who met the following inclusion criteria: employed as a master's prepared advanced practice nurse with at least 1 year of experience in the APN role. At least 1 year of APN experience is needed to define and implement all APN role dimensions, including leadership (Baker, 1987; Hamric & Spross, 1983). Maximum variation was used to enable the investigation of the phenomenon from a variety of diverse sources (Sandelowski, 1995). Data redundancy was achieved with 11 participant interviews completed; however, an additional three interviews were conducted to ensure no new themes emerged from the data (Morse, 2000; Sandelowski, 1995).

Data were collected using semi‐structured interviews and document analysis from March 2013–January 2014. Each participant completed an initial and follow‐up interview. The interviews were conducted using a guide developed by the research team and informed by the LEADS Framework. The study interview guide included 11 questions and was piloted by an advanced practice nurse not recruited for the study. The initial interviews lasted 60–90 min and were audio‐recorded and transcribed verbatim. Participants completed a brief demographic questionnaire at the conclusion of the initial interview. In addition to the interview data, two advanced practice nurse job profiles and three advanced practice nurse job postings were included in the analysis.

The initial findings of this study were shared with all 14 participants during the second interview. The themes and leadership capability domains were validated and further refined by the participants.

### 2.3 Analysis

The data were managed using NVivo10 (QSR International Pty Ltd., 2012) and analysed using qualitative content analysis (Attride‐Stirling, 2001; Graneheim & Lundman, 2004; Neergaard, Olesen, Andersenm, & Sondergarrd, 2009). To ensure study rigour, several processes for trustworthiness were included (Lincoln & Guba, 1985). The second interview with study participants allowed for member‐checking and feedback on the emerging data analysis. The primary researcher reviewed and coded all transcripts and one other member of the research team reviewed and coded 15% of transcripts. The research team reviewed and provided feedback on the coding framework and development of themes.

### 2.4 Ethics

Ethical approval for this study was obtained from the Research Ethics Boards at the two participating tertiary hospitals and by a hospital affiliated university Research Ethics Board.

## 3 RESULTS

As Table 1 shows, the all‐female sample was experienced and included fourteen advanced practice nurses. All participants noted leadership to be an expectation of their APN role.

**Table 1:** Study participant demographics

Demographic information	Number (*N *=* *14)	Percentages
Advanced practice nurses	14	100.00
Participants: Less than or equal to 49 years of age: Greater than 50 years of age (one participantchose not to disclose their age):	7 6	50.00 42.38
6–25 years of nursing experience: Greater than 25 years Years of nursing experience (one participantchose not to disclose their years of experience):	4 9	30.77 69.23
Less than 10 years of APN role experience: Ten or more years of APN experience:	5 9	35.71 64.29

### 3.1 Advanced practice nurse leadership

**Figure 1:**
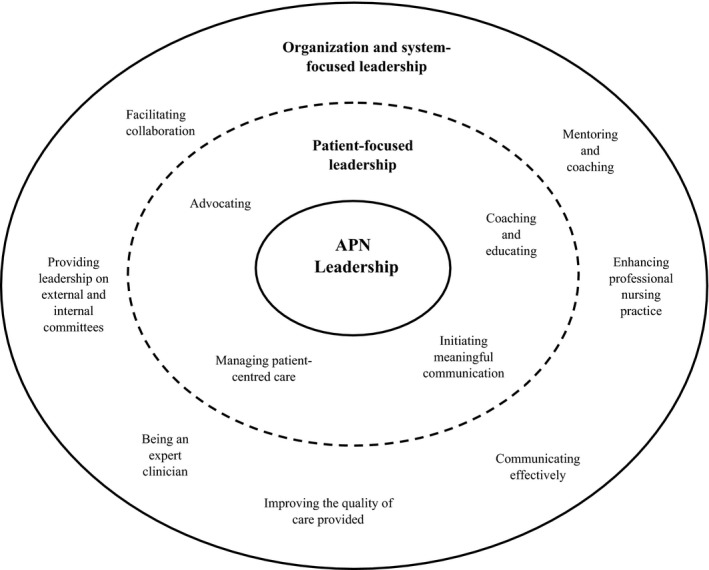
APN leadership capabilities model. APN, Advanced nursing practice

In this study, two overarching themes describing APN leadership were identified: “patient‐focused leadership” and “organization and system‐focused leadership”. Patient‐focused leadership as described by advanced practice nurses includes capabilities that are intended to have a direct impact on patients and families. “Organization and system‐focused leadership” includes capabilities that are intended to have a direct impact on nurses and other healthcare providers, the organization or larger healthcare system. Figure 1 summarizes the leadership themes and capabilities domains; APN Leadership Capabilities Model.

“Patient‐focused leadership” included the following four leadership capability domains: 1) managing patient‐centred care; 2) coaching and educating; 3) advocating and 4) initiating meaningful communication. “Organization and system‐focused leadership” included the following seven leadership capability domains: 1) improving the quality of care provided; 2) enhancing professional nursing practice; 3) being an expert clinician; 4) communicating effectively; 5) mentoring and coaching; 6) providing leadership on internal and external committees and 7) facilitating collaboration. Each capability domain has associated leadership capabilities. Leadership capabilities are the skills and abilities advanced practice nurses described as necessary for demonstrating leadership. Table 2 clearly defines the five “patient‐focused leadership” capability domains and provides supporting data for each capability. Table 3 defines the seven “organization and system‐focused leadership” capabilities domains and provides supporting data for each capability.

**Table 2:** Patient‐focused leadership capability domains, capabilities and supporting data

Patient‐focused leadership capability domain and definitions	Capabilities	Examples of data that fed into the capability
1. Managing patient‐centred care: using their clinical expertise in combination with advanced nursing knowledge to provide appropriate, high quality patient‐centred care to patients and families.	1.1 Providing clinical expertise in a specialty area—having specialized clinical expertise that is refined to meet the unique needs of the population they serve	I collected some data, I see less patients than physicians but my patients are generally sicker on average. My bounce back rates are lower and satisfaction rates are high, patients really appreciate the care. The function of the specialty clinic is to rapidly assess and manage patients. The wait time for a specialist physician is 6 months. They can not see patients on a return basis every week and every 2 weeks. Whereas in the specialty clinic as an advanced practice nurse, you can. The goal is to prevent admission to hospital. We know that when patients with this diagnosis have to go to the emergency department with symptoms, they often get a scan. With urgent access to the advanced practice nurse, not only have we avoided … most likely avoided a scan.
	1.2 Leading teams—taking on a leading role within the healthcare team	It is being able to work collaboratively with the team, recognizing everyone's skills and contributions…. But there is the small percentage of the time when its acute … and you need to step up and say “I am in charge and this is what we are doing. …”. Part of leadership is being central in the teams, working side by side with the nurses and the physicians, and building credibility and respect, and understanding where they are coming from as well as the patients.
	1.3 Promoting goal‐oriented care—focusing on achieving the healthiest outcomes for patients and families	…We are all trying to do the same thing, we have the same goals, is to make sure that the patient goes to the right place, at the right time, have the right resources in place, if possible. From the perspective of complex patients, you're always looking at the healthiest outcomes…That somebody with this diagnosis, how do we navigate them through what care be a very scary and puzzling care experience? To come through and have the best possible outcome.
	1.4 Using system level knowledge—understanding of how the healthcare system operates, who and what is involved at different levels and moving patients through the system was described by all participants as necessary for creating a seamless care experience for patients	People report we (advanced practice nurses) approach things differently—systems, big global picture, better communication, better ability to sort of understand where everyone is coming from. You just have the ability to identify what the patient needs with the patient, and ensure that those needs are being addressed. And speak to those needs when collaborating with other team members. We have the ability to recognize the gaps because we have a full sense of how the whole system integrates. We speak the language of every discipline. I understand that in order for the patient to seamlessly move through the system, they need somebody who understands the entire system and just not the clinical care. … I help patients with any particular issue related to their disease conditions. But also I help them with the bigger picture.
2. Coaching and educating—fostering trusting relationships and capitalizing on teachable moments. The two capabilities of this domain are	2.1 Facilitating independence and autonomy—capitalizing on opportunities to educate patients and families about disease processes, medications and new therapies	It is really about empowering and the focus is always on facilitating as much independence and autonomy as possible … Because that is what you need. You need the person to sort, be at a point to take charge. One of the things we do here, and maybe I am off, but that with a patient when we are mentoring, we help patients understand what the care should be, so that their expectation of what happen in care …
	2.2 Listening and explaining—Taking the time to listen to patients and clearly explain a diagnosis or a new treatment is crucial for patient acquisition of skills for self‐management	Part of my leadership on an in‐patient unit is taking care a step beyond routine standard level of care. Explaining things, listening, advocating for them a bit more. … So hopefully, it improves their outcomes or at least their satisfaction. The ability for a patient to better self‐manage their care through, enhanced education. The ability to spend more time with the client or the patient enables the patient to learn more about their illness or their whatever.
3. Advocating—being a patient advocate	3.1 Being a strong voice, negotiating on their behalf—representing and voicing patient and family needs at different forums and by assisting patients and families to feel safe and that the system is meeting their needs	I also advocate for the bigger community group. For example, we brought two new drugs for the treatment of this disease. The provincial government was going to put them on the formulary but they need help. I help them, because I know what I want for these patients and what would be best for them without any boundaries. The amount of advocacy and negotiation work we do with, you know, Pharmacare, Social Services, Canada Pension and Disability, insurance companies, other healthcare providers, you know its ongoing. One of the challenges is to keep the population in the eyes of the administration because it becomes nebulous. … care for this population, care for that population is a little different than another population. We do not always have leadership now that have an understanding, even in the population. Therefore, it is our responsibility to be always communicating and talking, making sure all of the needs and perspectives of the population come forward to the different forms.
4. Initiating meaningful communication—communicating with patients regardless of circumstances	4.1 Addressing the uncomfortable topics—initiating and addressing the more challenging topics and the tough conversations is perceived to positively impact patients and families, because they are able to talk about uncomfortable, and difficult elements of their care	We do know that our patients who see us are more comfortable discussing psychosocial and sexuality issues with us than they are with their specialist. They really appreciate the advanced practice nurse support and that holistic focus, and they often allow you to take the lead. They are not as comfortable as the advanced practice nurse in dealing with palliation. There was a complex patient that we had followed. We met with the team and they noted we weren't needed anymore. I am trying to get our team to go up and meet with the patient and family and say this is why we are no long involved in your care. We need to document it and we need to communicate that with the family because that's not fair to the family … that family deserves to understand from us why we're no longer involved … There is an accountability I feel is important.

**Table 3:** Organization and system‐focused leadership capability domains, capabilities and supporting data

Organization/system‐focused leadership capability domain and definition	Capabilities	Examples of data that fed into the capability
1. Improving the quality of care provided—using advanced nursing knowledge and clinical expertise to facilitate changes that improve the quality of care provided to patients and families.	1.1 Identifying gaps and integrating knowledge of the patient population, team and system to find solutions—being able to see the larger picture including the gaps in care and services and by using their knowledge of and access to resources across the spectrum of health care to minimize the gaps	I started this working group with this group of nurses. … They were very much working in silos. By bringing them all together and now we meet and I chair it, we just talk and we just have like informal discussions and share ideas. I'm bringing them together and enabling them to talk and share ideas and now they are working on project together. It is knowing the population. Something we do well as advanced practice nurses is we synthesize stuff. We take from five or six different areas or roles or whatever, and we can pull it together. … The synthesis piece is where you break it down and build it again and make something new out of it, that's the piece advanced practice nurses do really well. When I started, it was there is no place to transition our patients to… I spoke with the managers, the directors. I spoke with the clinic. Then, we developed a committee, a transitions plan and a transition program. It is identifying that gap, getting all the players together and hopefully moving forward and achieving the outcome we want. Initially, we have no means in‐house of providing the medication. We had a snowstorm and the courier couldn't bring the medication. I walked through a snowstorm. Very quickly after I met with our team to change this. Now, we have temporary privileges and all of that's gone away. … Now I am working with our team, seeing how we can support the other regions.
	1.2 Leading by example—showing others how to see things differently and how to respond professionally to challenging patient or team situations	It is role‐modelling. Going out and modelling how would I do clinical care, how would I respond to this family, how would I respond to this patient? Because role‐modelling at this point is bigger than nursing. It is role‐modelling within the whole healthcare team. I have been able to change people's minds, like, we can send this person home yes. Or no, that person can't go home for all these reasons because they are just going to be back here. … One of the quality things I do is follow‐up with people who leave without being seen. It's like a safety net. … I have caught a few significant things and brought them back in. … This has really changed people's perceptions of if this person has come in for 3 days in a row, there's something wrong with them. This is just not, you know, they can not get in to see their family doctor.
	1.3 Scanning the environment for best practices and ideas, creating and implementing change—being current and connected in order to facilitate practice improvements which lead to safe, timely, high quality patient care	I am creating practice guidelines for how we take care of patients with this diagnosis, …. to improve care. I knew I wanted the nurse practitioner role developed before I even got into the program. I had the discussion with the key people. So, there was an awareness of my plans. The director and physician lead, were just incredibly supportive and knew that was going to happen. And just the right people at the right time knew it needed to happen. I used the PEPPA framework to develop the role. So, we had a multidisciplinary collaborative approach to deciding what the role could look like, ensuring people were educated on what an nurse practitioner could and could not do and where they could and couldn't work. So, it was a methodical well strategized roll out of the role. What we do know is through the establishment of our advanced practice nurses led urgent access clinic, clinic in the community, telephone triage and telephone helpline, we have reduced hospital admissions and emergency room visits.
	1.4 Generating and using evidence in practice—creating, using and applying evidence to practice for the purposes of optimal patient care	Research is part of everything I do. Not only from conduct but also from the dissemination and also helping others and mentoring others to be able to understand research findings, evidence‐based practice. It is being an evidence expert in the sense of knowing the evidence and bringing that to the bedside in whatever way. It is working with our professional practice groups to develop research questions that we look at trying to change practice. … It's getting staff interested in that fact that research can be a simply good clinical question which you look at the evidence.
2. Enhancing professional nursing practice—bringing a high level of professionalism to their nursing practice	2.1 Advocating for nursing—being a strong and supportive voice for all nurses	I speak up for nursing. I always bring the nursing point of view to the table where there may be other disciplines. … I am always trying to promote the advanced practice nurse role. So that, people feel comfortable with the role and understand everything that we do. I think as a leader in a leadership role, that has to be part of my role, I want those nurses to be fully functioning and safe and good practitioners. … a larger part of my role is promoting and supporting nurses. I have an invested interest in the specialty, and I'm trying to promote nursing. I think advanced practice nurses have a role in that, in promoting nursing. And in saying it is good to be a nurse. This is why I am a nurse.
	2.2 Modelling professionalism—being a role model, so others understand what is expected as a nurse and a professional	I think you learn what nursing is at school but very quickly you're socialized a totally different way once you get into a new culture. So that when you talk to people, they do remember back to what they went into nursing for and what they were going to focus on. And then it gets quickly pounded down. That is what I want to pull out, ignite the spark. Leadership for me is active. It is demonstrating constantly what it means to be a professional nurse. … I think it's to give those new nurses grounding and give those new nurses a sense of, that we care about them and their professional development, and our expectations as an organization. … That they are coming in and they are knowing that at our organization, we are expected to practice in a certain way. And there is a high standard for that practice.
	2.3 Providing informal bedside teaching—taking every opportunity to share their knowledge and teach nurses as well as other health care	They look to me every day for answers to questions, clinical questions. I think that being and advanced practice nurse, you have a level of knowledge that nurses acknowledge, appreciate and want to learn from. It is bedside leadership, bedside education … the onus is on advanced practice nurses to create nurse leaders at the frontline.
	2.4 Visioning—seeing what nursing can be and helping nurses and the profession grow and advance	It is very much to lead nursing. … I think you need to be able to stimulate passion, stimulate excellence, stimulate ongoing learning, stimulate the desire to grow as a nurse, provide holistic care … So much of it is facilitating, seeing where we are, where do we need to go, and then what do we do to get there? It's either me or who else that would do that.
3. Mentoring and coaching—fostering trusting relationships with the goal of building capacity within others	3.1 Engaging others—having an encouraging approach and a demeanour that other people respect and learn from	Leadership is not just being at the forefront of teaching and leading and showing by example. It is encouraging other staff members. That you can take the lead, you can be a leader, and here is how you can do it. I am right behind you. You are working under the same principles that I am. You can make you are way and form a bit of an autonomous practice for yourself if you take these things on. In a very diplomatic way and make sure you've got all the team on side and you follow your principles and you don't go outside your scope. The staff is very comfortable coming to talk to me about lots of things. It could be interactions on the units, bedside clinical things. “How do you?” … I do a lot of coaching as to what might you say, how to you approach a situation. I think that you need to be able to stimulate passion, stimulate excellence, stimulate ongoing learning, stimulate the desire to grow as a nurse, to provide holistic care, to …
	3.2 Being a mentor—assisting nurses and colleagues to develop new skills and inspire them to work towards their professional goals	I lead and guide and sort of hope to work myself out of a job by building competence and building skills, and developing that in other nurses and other healthcare providers. … So even a couple of years ago, another advanced practice nurse and I worked with a team, to help the nurses work to full scope. I see leadership as a way that you're kind of guiding and mentoring other nurses and healthcare providers to push the practice forward and feel confident and able. It is important to mentor new staff in professional nursing, what it means to be a professional nurse, and not to fall into sort of the norm, the status quo, to think and to be the kind of nurse that you would want to have caring for your family member. So leadership for me is active. It is constantly demonstrating what it means to be a professional nurse. I have been very fortunate with tremendous mentorship and support. It is all about passing it on and helping others to do that.
4. Being and expert clinician—as having extensive knowledge of the patient population and a proven ability to translate that knowledge to improve care	4.1 Establishing clinical credibility—being respected for their knowledge of the patient population and nursing	My power is not formal. It is built on my ability to prove my credibility and to be able to somehow insert myself at key positions and key ears to sort of say we should be doing this… I chaired the committee. I did that for a term and am still one of the committees, and I am providing leadership. We are pushing address with initiative to address a particular disease. We'll be meeting with the government officials on those initiatives. It means being a respected stakeholder in important things that are disease‐specific. Leadership is when people look at you as an expert. You know, have respect for you because of your clinical judgment or just knowledge base in general. … I think being an advanced practice nurse catapults that a bit more … That in itself puts me in a leadership position that I have to kind of be mindful of at all times.
	4.2 Formal teaching of nursing and health professional students—teaching others in a formal setting	With respect to formal teaching … I still do, I would say 95% of all the nursing care orientation to the nursing staff coming into our area with respect to my area of expertise. I continue some formal teaching on sort of my research findings but also teach in the fellowship program through the department because I have a cross appointment there. I have gone off to do a lot of education. People have called a lot of its with certain communities … Then, the next thing I find myself driving to the community to give a half‐day workshop… The word gets out. And I would never say I am an expert. So much of my leadership very much depends on the networks that I make.
	4.3 Having confidence in one's self and as a practitioner—being confident in one's self as a person and as a nurse in order to lead	When we started, it was not about sort of what all you were doing, it was actually who you were. There was a lot of give and take about expectations, role development and role implementation. We had tremendous support in which … I think we led that as well. Fortunately, we are all very senior nurses and had a lot of experience, and were not afraid to be our own voice, advocate and leader. I think advanced practice nurses, need experience for sure. That comes with time. They need to have confidence in their ability to be a leader, confidence in their ability to make decisions that are, you know, what is right for the patient, the staff, the department, whatever it is. So confidence, knowledge. You obviously need to have a strong knowledge base as well. It just can't be in name only. It has to be in action as well.
5. Communicating effectively—listening and expressing one's opinions and ideas in a constructive and productive way	5.1 Demonstrating effective communication—being able to actively listen and engage in conversations with purpose	Leadership in a lot of ways is just sitting and listening and saying, ok you know, these are your ideas, what are you going to do about them, and how can I help you get there? And it has been amazing, if you just give them a little push, it is so gratifying. I think the ability to communicate effectively stems not only from experience and knowledge but it's a mutual trust that you have with your colleagues that says, this is somebody who should be listened to. It is really being able to step out of your comfort zone, do the hard work or ask the hard questions that everybody in the room is thinking and nobody wants to say. Being willing to put your head above the parapet and say, but this is not acceptable.
	5.2 Understanding your audience—understanding who you are speaking with, and their goals or agenda.	Feeling like an informal nurse leader and breaking through the physician barrier of like, I need your buy in but it's going to happen, but I still need your buy in. Just knowing the right way to go up the ladder. You have to be careful with how you talk, and you have to be careful with what your message is. Your intended message, your intended audience. Sometimes you have to be very political in how that's delivered. What I learned is you have to speak the language of the individual, find out what the individual is or the groups are interested in, and try to meld your agenda or your patient's agenda in with that. … Being mindful of how I communicate as well as ensuring that you're communicating with the appropriate stakeholders and players involved in whatever you're doing.
6. Providing leadership on internal and external committees—taking an active leadership role as chair, co‐chair or executive position on committees within the organization where they work as well as external organizations	6.1 Chairing or holding an executive positions—having a leadership role on an internal and or external committee	It is important that you stay involved in your specialty associations. You need to be a leader on working groups. You need to be at conferences and doing things. I sit on the Canadian Association of this specialty Nurses. In the last few years, I have helped write standards because there were never any. I first wrote competencies. Then, we wrote standards for the population. The competencies are being used by an academic centre as a credit course. I am chair of the nurse special interest group of the Canadian specialty Society. So that's a national level of taking a different type of leadership. And then several committees more nationally and internationally.
7. Facilitating collaboration—developing effective partnerships to work towards a common goal	7.1 Engaging other people and or organizations to purposefully work as a team—bringing people together to tackle an issue and improve patient care	We pulled together and started working with all the stakeholders. So key champions, all the staff, administration, and the hospital. We did a hospital wide policy because that was needed. … We were getting quite a few of these patients, there was a lot of stigmatizing and marginalization. That's how I got into it. I thought, I need to know more—who is out there, who is working with them? That is how I connected with the teams in the community. I realized they were as hungry to do a connection liaison. Part of the work I did was to sort of start to bridgework, how would we look at this. We've developed a really nice collegial work. … We have built some really nice bridges with the teams and built supports. We developed a strategy… This is again where you build partnerships. I collaborate really well. I have formed really good relationships with people in the community as well. Certain family doctors, the health team, the Community Centre. It is really important to, even in acute care, build these important community partnerships.

## 4 DISCUSSION

The APN Leadership Capabilities Model (Figure 1) and associated domains (Tables 2 & 3) illustrate the nature of APN leadership and the complexity of APN roles. Each domain includes one or more capabilities. The model creates a language and a road map from which advanced practice nurses and others can begin to articulate their leadership roles. Nurses in advanced practice roles must be able to clearly articulate their roles to operationalize the full range of their capabilities (Calkin, 1984; Jones Charbachi, Williams, & McCormack, 2012).

Leadership is often referred to as a competency in APN literature (CNA, 2010, 2014; Elliot et al., 2012; Hanson & Spross, 2005). The accepted definition of competency is practicing to a minimum safe standard in a predictable environment (CNA, 2005; Dickson et al., 2007). The advanced practice nurses in this study described being leaders in dynamic, unpredictable settings that required their sophisticated nursing knowledge and expertise. The LEADS Framework is founded on the concept of leadership as a set of capabilities that enable leaders to have the necessary skills to thrive in complex environments such as those described by the advanced practice nurses in this study (Dickson, 2010). Using the LEADS Framework to inform this study enabled the findings to be more representative of the reality of APN leadership as described by the participating advanced practice nurses.

The capabilities described in the APN Leadership Capabilities Model (Figure 1; Tables 2 & 3) are comparable to many of the capabilities found in the LEADS Framework, for example, both include the capability of communicating effectively. Similarly, advanced practice nurses in this study described “enhancing professional nursing practice—bringing a high level of professionalism to their nursing practice” through modelling professionalism and having a vision for nursing practice (Table 3), which is relevant to the LEADS capability of orient themselves strategically to the future (Dickson, 2010). One main difference between the LEADS framework and leadership described by advanced practice nurses in this study, is the focus of activities. When the advanced practice nurses described their leadership, it was in the context of making change at the level of the patient, nurse, healthcare provider or program and less frequently at the level of the entire healthcare system. The language used to describe the leadership capabilities in LEADS often refers to system level change and does not fully capture APN leadership.

Advanced practices nurses described their leadership as patient‐focused and organization and system‐focused. Leadership as described by Kouzes and Posner (2007) is a relationship between people. When the relationship is built on mutual trust, respect and confidence exemplary outcomes can be achieved. To build the relationship described above, Kouzes and Posner (2007) described five practices and ten commitments a leader can develop and practice to be exemplary. There are some common themes found between the practices and commitments of *Exemplary Leadership* (Kouzes & Posner, 2007) and the APN Leadership Capabilities Model (Figure 1). For example, advanced practice nurses described modelling professionalism comparable to *model the way* a practice of Exemplary Leadership. Kouzes and Posner (2007) Exemplary Leadership includes the practice of *encourage the heart*, which is described as positive reinforcement of achievements and excellence. Advanced practice nurses in this study did not describe celebrating their successes as part of their leadership; therefore, a similar capability to the practice of *encourage the heart*, is not found the APN Leadership Capabilities Model.

The leadership capabilities outlined in the APN Leadership Capabilities Model (Figure 1) are comparable to several competencies found in other APN frameworks. System leadership is one of the core competencies found in the *Pan‐Canadian Core Competencies for the Clinical Nurse Specialist* (CNA, 2014). It includes competencies such as facilitating interprofessional collaboration and integrating knowledge to make change and many others. Leadership is also a core competency in the *Canadian Nurse Practitioner Core Competency Framework,* which includes competencies such as managing clinical teams and acting as a mentor and preceptor (CNA, 2010).

Similarities can also be seen between the APN Leadership Capabilities Model and the *Core Competencies of Advanced Practice Nursing* model (Hamric, Spross & Hanson, 2009). Both models have layers of competencies or capabilities arranged in a circular manner, emphasizing patient care as the central feature of APN. Hamric, et al. (2009) denotes clinical and professional leadership as a core competency of APN practice. The clinical and professional leadership skills they describe are similar to some of the capabilities described in *patient‐focused* (Table 2) *and organization and system‐focused leadership* (Table 3).

Clinical and professional leadership activities were also identified by Elliot et al. (2012) in a large Irish study that was the first to examine APN leadership. For example, Elliot et al. (2012 pg. 1040–1046) described the following clinical leadership activities: *initiates and changes patient/client care through practice development; changes clinical practice through formal education; guides and co‐ordinates the activities of the multidisciplinary team; and mentors and coaching the multidisciplinary team in clinical practice. These activities are comparable to the following capability domains in our* study: improving the quality of care provided; managing patient‐centred care; and mentoring and coaching. Elliot et al. (2012) also described professional leadership activities that are similar to the leadership capabilities domains described by participants in our study such as: engages in professional organizations at a national or international level. These activities are relevant to the “organization and system‐focused leadership” capability domain of providing leadership on external committees and bringing the knowledge back to their practice environments. There were also some differences in the results between the two studies including the organizing themes as well as some specific leadership activities/capabilities identified in one but not the other study. Whereas Elliot et al. (2012) identified APN leadership as clinical or professional leadership activities, in the APN Leadership Capabilities Model leadership is defined through a set of capabilities that have an impact on the patient and the system or organization. Capabilities as defined by Dickson (2010) in LEADS are actions enacted in a complex, ever changing environment for which the person has more than the minimum knowledge and skill to act safely. An advantage of examining leadership in the context of patient, organization and system factors is that it provides a framework through which to begin to articulate the outcomes of APN leadership and understand the complexity of advanced practice nurses actions as leaders. Some of the differences in findings may be related to methodology. Elliot et al. (2012) national study used a case study method that included non‐participant observation of Clinical Nurse Specialists and Advanced Practitioners (Clinical Midwife Specialists and Advanced Nurse Practitioners), interviews with administrators and clinician team members and document analysis. Our study was much smaller in scope, involving only acute care advanced practice nurses in one Canadian city. Differences may also potentially be related to contextual differences in the study settings (country), healthcare systems, education of practitioners and or APN role expectations.

Leadership is a shared responsibility of all nursing roles (CNA, 2009). For graduate prepared nurses working in APN roles, leadership is a core competency not simply a shared responsibility (CNA, 2008, 2010, 2014; ICN, 2009). The leadership skills of Registered Nurses working in non‐advanced practice roles (such as staff nurses) are often defined as the “staff nurse behaviours that provide direction and support to clients and the healthcare team in the delivery of patient care” (Patrick, Spence Lanchinger, Wong, & Finegan, 2011; pg 450). This definition has similarities to how advanced practice nurses in this study described their *patient‐focused* leadership capabilities in that both are focused on the best outcomes for the patient and family. The difference, noted by Patrick et al., (2011) and described by the advanced practices nurses in this study, is that advanced practice nurses demonstrate their leadership through integration of these capabilities in complex and unpredictable care environments.

Leadership in the context of APN has varying definitions; often these definitions differentiate APN leadership as either clinical leadership, which influences patient care at the local level, or professional leadership, which involves a broader context of national and international organizations (Carryer, Gardner, Dunn, & Gardner, 2007; Elliot et al., 2012; Fealy et al., 2011; Gardner, Chang, & Duffield, 2007; Hamric et al., 2009; Hanson & Spross, 2005; Mantzoukas & Watkinson, 2007). Categorizing APN leadership as, clinical and professional leadership, did not resonate with participants of this study, they preferred “patient‐focused and organization and system‐focused leadership”. The themes and leadership capabilities in the APN Leadership Capabilities Model captured and provided descriptive language to articulate the breadth of leadership provided by advanced practice nurses.

Leadership is a skill that must be developed, practiced and coached (Dickson, 2010; Kouzes & Posner, 2007). LEADS is a leadership framework intended to be a guide for developing leadership capabilities to be an effective leader, regardless of position or title in health care (Dickson, 2010). The LEADS Framework has informed this study and its findings. Educators could adopt the APN Leadership Capabilities Model as a guide to developing leadership capabilities in advanced practice nurses.

Leadership is embedded in a series of complex actions in care delivery and may not be apparent or tangible (Calkin, 1984; Whitehead et al., 2017). As a result, the contribution that advanced practice nurses make to the healthcare system is often lost because what they do is considered clinical practice and not leadership. Beginning to use the language provided in “patient‐focused leadership and organization and system‐focused leadership” can assist advanced practice nurses in articulating what they do. This will allow those in decision‐making roles to better understand and capitalize on the valuable resource of APN leadership and what it contributes to patient, organizational and system outcomes.

Other studies have shown that for many hospital decision‐makers, the most common avenue for them to receive information about APN roles is through advanced practice nurses in their organization (Carter et al., 2013). Therefore, it is incumbent on advanced practice nurses to be able to describe their roles and educate key stakeholders. Having a collaborative, evidence‐based APN vision and framework specific to the organization could facilitate consistency in communication of APN roles and could assist physicians and administrators to better appreciate the complexity and value added of APN roles. It is equally important for administrators and other stakeholders to educate themselves about APN roles.

The findings of this study advance understanding and provide a descriptive APN leadership model that could contribute to an overall vision and framework for APN practice in an organization. Supportive administrators (nursing and medical) are integral to implementing and sustaining APN roles. Therefore, the more consistent and evidence‐based the information that decision‐makers receive about APN roles, the more likely they are to understand and support these roles (Carter et al., 2010, 2013; DiCenso et al., 2010).

APN leadership needs to be articulated clearly, so that the impact of this leadership can be understood and measured (Kilpatrick, Kaasalainen, et al., 2014; Kleinpell, 2013). Unfortunately, when the abilities of professionals are not understood, they are often underused and talent can be wasted and the potential benefits to patients, nurses, healthcare providers and the larger health system can be lost.

### 4.1 Study limitations

The study focused on describing leadership from the perspective of a sample of advanced practice nurses in two hospitals in one Canadian province, which may minimize the transferability of these results, given the variation in advance practice nurse roles across the globe. Although the study was open to both female and male advanced practice nurses in the two study sites, only females chose to participate. There may be gender differences in how leadership capabilities are defined and enacted that were not captured in this study. Future studies are required to expand on these findings with a larger sample spanning other practice settings, for example, primary health care.

## 5 CONCLUSION

This qualitative descriptive study provides evidence to better understand and use leadership capabilities as a lesser known dimension of APN roles. Having language that describes APN leadership can enable advanced practice nurses to recognize the leadership capabilities they demonstrate as part of their daily practice. The ability to recognize and then describe their leadership will enable advanced practice nurses to clearly articulate their leadership to decision‐makers, other healthcare professionals and the public. This may enhance understanding of the APN role and contribute to optimization of APN leadership capabilities. The current state of health care is one of continuous changes where leaders at all levels are needed to lead changes to create positive results. This study describes APN leadership and the contributions APN leadership make to patient, organizational and system goals.

## CONFLICT OF INTEREST

None declared.

## AUTHOR CONTRIBUTIONS

All authors have agreed to the final version.
